# Effects of maize (*Zea mays*) genotypes and microbial sources in shaping fall armyworm (*Spodoptera frugiperda*) gut bacterial communities

**DOI:** 10.1038/s41598-021-83497-2

**Published:** 2021-02-24

**Authors:** Charles J. Mason, Kelli Hoover, Gary W. Felton

**Affiliations:** Department of Entomology, The Pennsylvania State University, University Park, PA 16802 USA

**Keywords:** Microbial ecology, Symbiosis

## Abstract

Plants can have fundamental roles in shaping bacterial communities associated with insect herbivores. For larval lepidopterans (caterpillars), diet has been shown to be a driving force shaping gut microbial communities, where the gut microbiome of insects feeding on different plant species and genotypes can vary in composition and diversity. In this study, we aimed to better understand the roles of plant genotypes, sources of microbiota, and the host gut environment in structuring bacterial communities. We used multiple maize genotypes and fall armyworm (*Spodoptera frugiperda*) larvae as models to parse these drivers. We performed a series of experiments using axenic larvae that received a mixed microbial community prepared from frass from larvae that consumed field-grown maize. The new larval recipients were then provided different maize genotypes that were gamma-irradiated to minimize bacteria coming from the plant during feeding. For field-collected maize, there were no differences in community structure, but we did observe differences in gut community membership. In the controlled experiment, the microbial inoculation source, plant genotype, and their interactions impacted the membership and structure of gut bacterial communities. Compared to axenic larvae, fall armyworm larvae that received frass inoculum experienced reduced growth. Our results document the role of microbial sources and plant genotypes in contributing to variation in gut bacterial communities in herbivorous larvae. While more research is needed to shed light on the mechanisms driving this variation, these results provide a method for incorporating greater gut bacterial community complexity into laboratory-reared larvae.

## Introduction

Insect herbivores commonly harbor diverse microbial assemblages in their gut tissues^1–3^. In some instances, plant diets can contribute substantially to gut microbial community taxonomic and functional composition^4–6^. This is especially the case for lepidopteran herbivores, where variable patterns of bacterial gut colonization have been observed in response to different host plant species, genotypes, and ontogeny^7–15^. The community membership and composition that ultimately resides in lepidopteran gut tissues can be affected by several factors related to the host insect ecology and gut biochemistry, the phytochemistry of ingested plant foliage, and the source of bacteria that seed the gut bacterial community. These complex, multipartite interactions are generally not-well elucidated for larval lepidopterans, and whether there are overarching effects of any one of these processes on components of gut microbial communities is unclear.

Fundamentally, the variation observed in lepidopteran gut microbial communities can generate conceptual and technical hurdles to studying microbial functions in plant–insect interactions. This is especially the case in the laboratory where the microbial communities harbored by lepidopterans are often highly simplistic compared to those collected from the field^16,17^. One method to increase gut microbial diversity under laboratory conditions is through introduction of microbial assemblages from larval feces or gut contents to germ-free recipients. Fecal transplant methods are often employed in studies on vertebrates^18–20^ and experimental transplant approaches using gut contents has also been used for some groups of insects^21^. In nature, transfer of microbes between conspecifics and across generations through feces and oral trophallaxis can occur in several insect orders^22–24^, but is not a described process in lepidopterans.

The principal aim of our study was to adapt fecal transfer methods for a foliage feeding lepidopteran to understand how complex gut communities respond to the folivore’s digestive system when feeding on different plant genotypes. Currently, there is circumstantial evidence that plant-associated bacteria contribute significantly to bacterial diversity in herbivore guts^8,12,25,26^. However, we do not know if this variation is driven by constant replacement of bacteria from the environment or if initial establishment of gut bacteria dictates community composition throughout the larval stages. Identification of resident components of the bacterial community and impacts related to changes in plant genotypes may better elucidate functionally important insect–plant–microbe interactions. Additionally, strategies to incorporate some of the microbial complexity observed in field-collected animals into laboratory systems is needed to better link lepidopteran model systems to field conditions.

Fall armyworm (*Spodoptera frugiperda*) is an important polyphagous lepidopteran^27,28^, which exhibits substantial variation in its microbial community. Primarily comprised of bacteria, the microbial community is fairly plastic and is affected by the ingestion of different diets^7^. Like other noctuids^10,11,17,29–31^, the microbial communities of fall armyworm larvae reared on artificial diets are often simplistic compared to field collections or when they are fed plant-based diets^7,16,32–34^. Many of the bacteria that populate the plant-fed fall armyworm gut (i.e., *Pseudomonas, Enterobacter, Pantoea, Klebsiella*)^7,32^ are associated with the foliage and soils^26,35–37^. However, gut bacteria colonizing fall armyworm midguts are not entirely transient and simply passing through the digestive tract, since single timepoint introductions of microbial populations in axenic larvae establish and proliferate throughout their development^38^. Unlike what has been described for other insect orders and species^1,3,5,6^, clear beneficial functions of gut microbes in fall armyworm larvae have not been determined. However, some bacterial taxa have been observed to suppress plant defenses^33^, while other isolates can exacerbate the deleterious effects of plant defenses on caterpillars^16^.

The main hypothesis of our study was that there would be an impact of host plant genotype on shaping the bacterial community of fall armyworm. We used a series of observations and manipulative experiments with fall armyworm and maize (*Zea mays*), one of fall armyworm’s major host plants. First, we evaluated how field-grown maize genotypes with different levels of resistance to fall armyworm influence the gut bacterial communities of larvae feeding on those hosts. Then, we performed fecal transplants from those larvae to new, axenic recipients to evaluate how different donor communities interact under more controlled conditions. The donor larvae were subjected to bioassays using maize leaves sterilized with gamma irradiation to evaluate if different components of the bacterial community are transient. The collective results from these experiments will provide better understanding of the microbial ecology of lepidopterans and the role of plants in mediating gut microbe interactions.

## Results

### Influence of field-grown maize on fall armyworm gut bacterial communities

**Figure 1:**
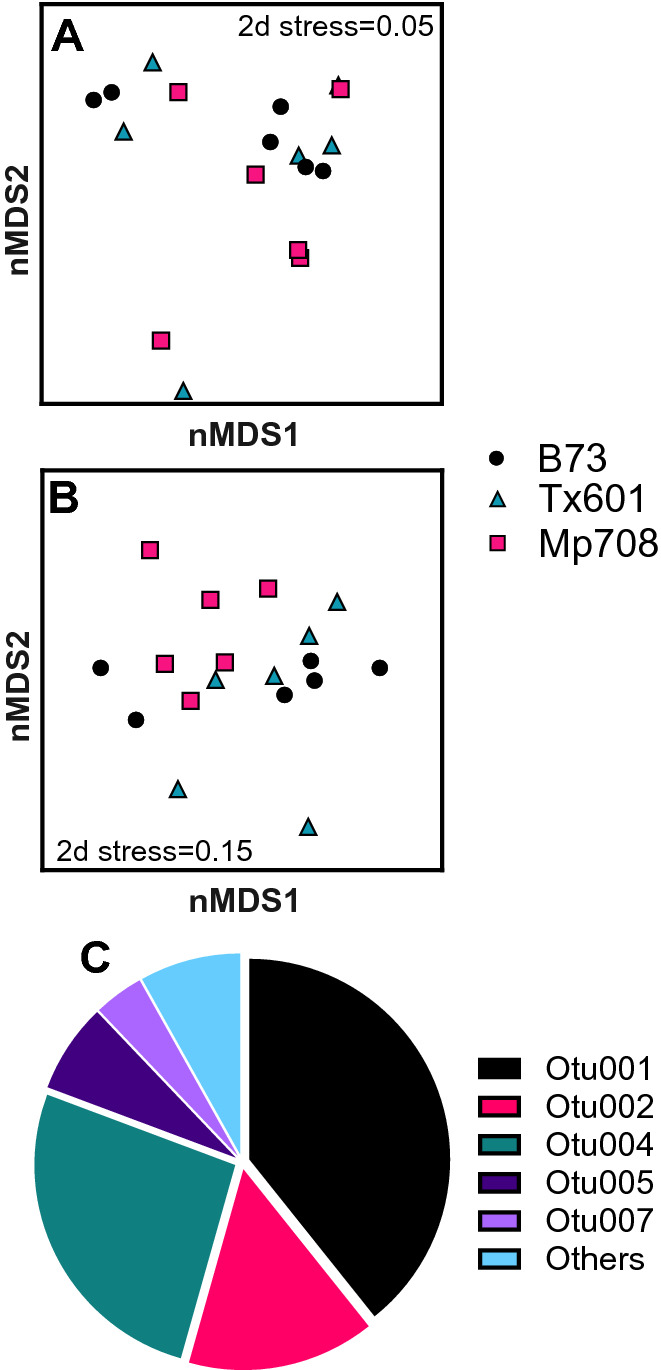
Influence of plant diet on fall armyworm gut microbiome structure (**A**), membership (**B**), and dominant members (**C**). There was no effect of different field-grown plants on the structure of OTUs, but there was an effect on community membership. Five OTUs comprised ~ 90% of the relative abundance across all treatments and were present in similar composition across treatments (Otu001: Unclassified Enterobacteriaceae; Otu002: *Enterococcus;* Otu004: *Pseudomonas*; Otu005: *Neorhizobium*; and Otu007: Unclassified Enterobacteriaceae). B73 and Tx601 are considered susceptible genotypes and Mp708 is resistant to fall armyworm.

When fall armyworm larvae were fed different maize genotypes, we observed differences between gut operational taxonomic unit (OTU) membership, but not in the structure of the bacterial communities (Fig. 1). Non-metric multidimensional scaling (NMDS) visualization using Bray–Curtis dissimilarities suggested minor differences in gut community structure (Fig. 1A). Permutation MANOVA analyses supported these trends in that different plant genotypes did not influence the structure as a whole (F_2,15_ = 0.72; p = 0.60). Differences emerged when we used Jaccard presence/absence dissimilarities to evaluate OTU membership (Fig. 1B), where the different maize genotypes caused insects to harbor different gut bacterial taxa (F_2,15_ = 1.29; p = 0.023). Conducting pairwise comparisons between the groups showed significant differences in Jaccard dissimilarities between the maize genotypes Mp708 and Tx601 (t_1,10_ = 1.15; p = 0.04) and between Mp708 and B73 (t_1,10_ = 1.21; p = 0.023), but not between B73 and Tx601 (t_1,10_ = 1.04; p = 0.234). Alpha diversity estimates were not influenced by different plant genotypes in the fall armyworm guts of larvae fed field-collected leaves (Supplemental Table 1).

The disconnect in plant genotype effects on structure and membership is largely due to five OTUs that across all samples comprised ~ 90% of the relative abundance of the OTUs (Fig. 1C). The most prevalent OTUs in the fall armyworm gut samples included: OTU 001 Unclassified Enterobacteriaceae (~ 50%), OTU 002: *Enterococcus* (~ 20%)*;* OTU 004: *Pseudomonas* (~ 20%); OTU 005: *Neorhizobium* (~ 10%); and OTU 007: Unclassified Enterobacteriaceae (~ %5). These individual OTUs were not significantly different between insects feeding on different maize genotypes (Supplemental Table 2).

Compared to the gut samples from larvae provided foliage from maize genotypes Tx601 and Mp708, the frass had a dramatically different OTU composition (Supplemental Figure 1). Frass had lower relative abundances of the five most prominent OTUs in the guts of those individuals. Alpha diversity metrics also supported these observations, where there was significantly greater OTU richness in the frass, increased estimates of diversity, and a more even distribution of OTUs (Supplemental Tables 3, 4).

### Effects of donor microbial sources and sterile plants on larval gut microbial communities

**Figure 2:**
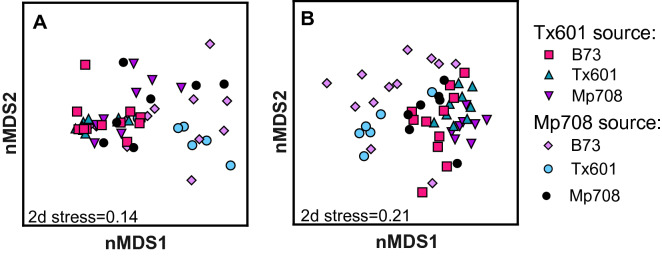
Non-metric multidimensional scaling of 16S-rRNA OTUs from guts of fall armyworm receiving transplants from donors that fed on maize genotypes Tx601 (susceptible) or Mp708 (resistant). Larvae were provided gamma-irradiated maize (see Fig. 3) and their gut microbiota were extracted and analyzed. Both Bray–Curtis (**A**) and Jaccard (**B**) distances were used to examine microbial structure and membership, respectively.

When axenic, recipient fall armyworm received the microbial community prepared from frass, both donor source and plant genotype impacted OTU membership and structure (Fig. 2). For Bray–Curtis dissimilarities, donor microbes (F_1,47_ = 16.2; p < 0.001), bioassay plant genotype (F_2,47_ = 2.1; p = 0.044), and their interaction (F_2,47_ = 2.92; p = 0.011) all influence the OTU membership in the fall armyworm gut (Fig. 2A). Similarly for Jaccard dissimilarities, we observed an influence of the donor microbes (F_1,47_ = 3.31; p < 0.001), bioassay genotype (F_2,47_ = 1.5; p < 0.001), and their interaction (F_2,47_ = 1.39; p < 0.001) on resulting OTUs in fall armyworm larvae (Fig. 2B).

Alpha diversity metrics followed varying trajectories (Supplemental Table 6). Bioassay plants did not have strong effects on the larval gut community alpha diversity. There was a significant effect of donor source on OTU richness (F_2,47_ = 7.25; p = 0.009), as well as Simpson (F_2,47_ = 4.48; p = 0.038) and Shannon metrics (F_2,47_ = 4.09; p = 0.048); larvae that received Mp708-donated microbes had greater OUT richness and diversity across all bioassay plants. Additionally, we observed statistically significant interactions between the donor source on OTU richness (F_2,47_ = 4.3; p = 0.009), Chao1 (F_2,47_ = 3.23; p = 0.049), Simpson (F_2,47_ = 5.93; p = 0.005), and Shannon metrics (F_2,47_ = 8.65; p < 0.001) (Supplemental Table 6).

**Figure 3:**
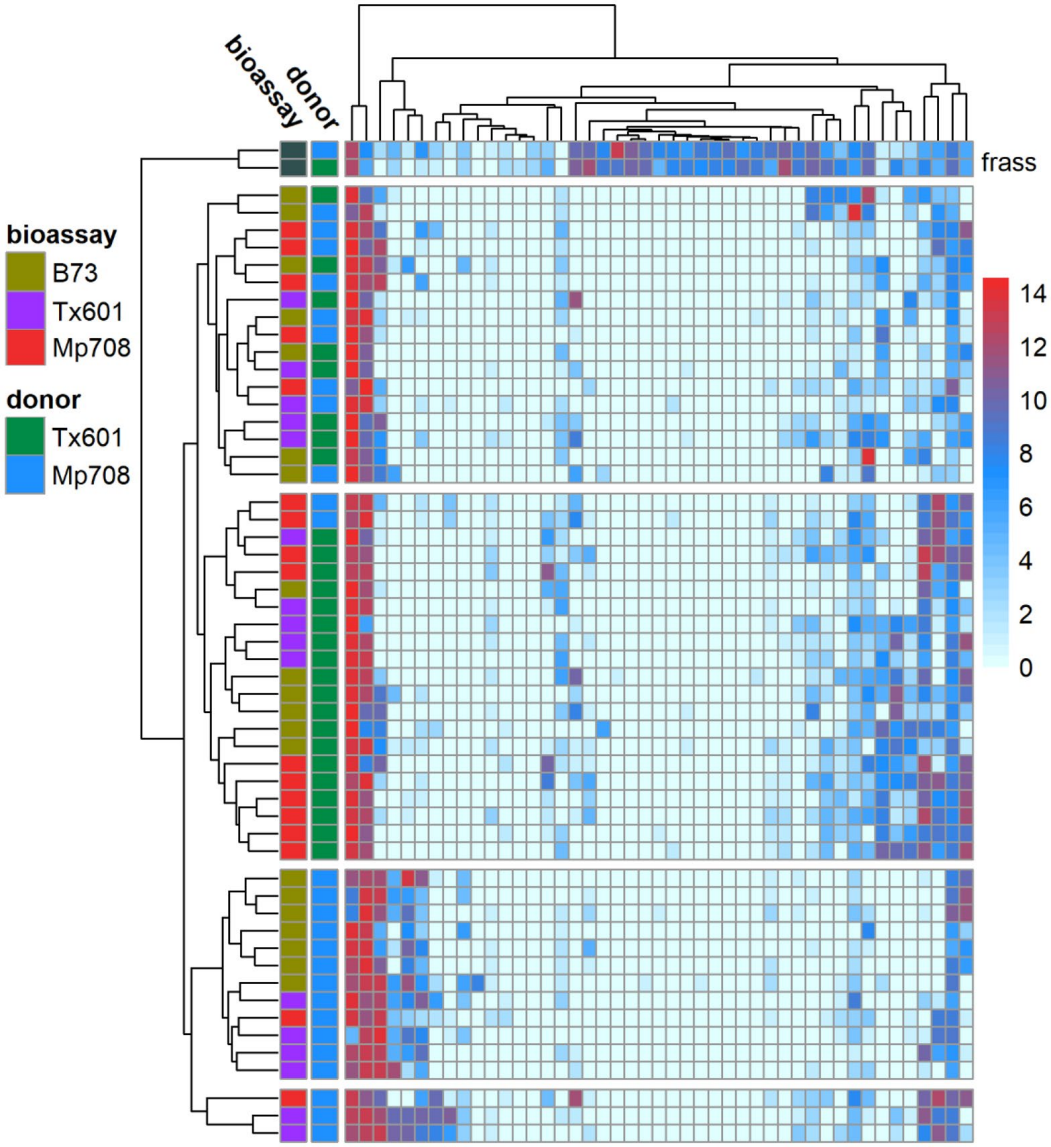
Heatmap showing abundance and distribution of the 100 most prevalent OTUs in fall armyworm guts. Donor indicates the feeding substrate of the fall armyworm larvae used to provide frass for transfer to recipients, while bioassay indicates the feeding substrate of the recipients. Clustering shows that the resulting microbes are influenced strongly by the donor and partially by the bioassay food substrate. The top cluster along the y-axis indicated by “F” are the pooled samples provided to the insects. The heatmap was constructed using log_2_(x + 1) transformed values.

**Figure 4:**
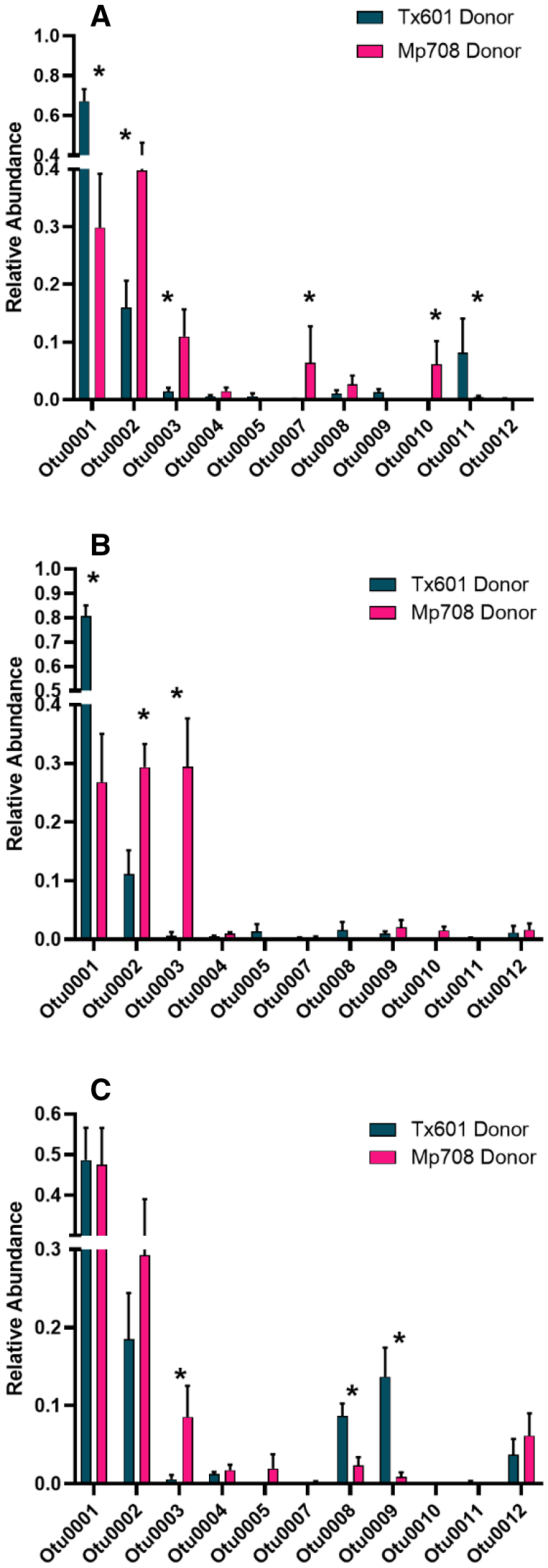
Relative abundance of more prevalent (> 97% of read relative abundance) OTUs from the gut of fall armyworm fed on susceptible B73 (**A**), susceptible Tx601 (**B**), and resistant Mp708 (**C**) maize foliage. Donor indicates the feeding substrate of the fall armyworm larvae that provided frass for transfer to recipients. Asterisks represent significant differences in OTU relative abundances.

The relative abundance of individual OTUs harbored by fall armyworm larvae differed between the inoculation source, bioassay plant, and their interactions (Fig. 3; Supplemental Table 7). The OTUs populating the guts of recipient larvae that received donor microbial communities exhibited some differences compared to those collected in the field (Fig. 4; Supplemental Table 8). Across all treatments, larvae receiving bacteria donated by Tx601 frass exhibited high relative abundances of OTU 001: Unclassified Enterobacteriaceae (66% of the reads) and OTU 002: *Enterococcus* (14%). Larvae receiving bacteria donated from Mp708 were slightly different than those receiving Tx601, with OTU 0001: Unclassified Enterobacteriaceae comprising 35% of the community and OTU 002: *Enterococcus* comprising 33%. One OTU that was present in significantly higher relative abundance in larvae receiving Mp708 donor frass was OTU 003: *Paenibacillus* (Supplemental Table 8). This OTU comprised 15% on average of the reads in this insect group, which was much greater than those that received Tx601 frass (0.1% of the reads) and those collected in the field (< 0.001% of the reads). For the group receiving Tx601 frass, OTUs classified as *Kaistia* (Otu008) and *Rhodococcus* (Otu009) comprised greater percentages of the relative abundances (3.5% and 5%, respectively) compared to those receiving Mp708 frass (1.8% and 0.8%, respectively). There were statistical interactions between inoculation source and bioassay plants (Supplemental Tables 7, 8). In many instances, individual OTUs responded differently between the inoculation sources and plant genotypes, with some increasing in relative abundance while others decreased (Supplemental Tables 5, 8).

While we observed differences in OTU relative abundances in the larval guts on susceptible maize (B73 and Tx601), larvae feeding on resistant Mp708 maize leaves were more similar (Fig. 4; Supplemental Table 9). When performing contrasts within the Mp708 bioassay treatment groups, the two most prevalent OTUs (OTU0001 & OTU0002) were not significantly different between the donor sources (p = 0.97 and p = 0.54, respectively). Of the most prevalent OTUs, there were three that differed between these sources: Otu0003: *Paenibacillus* was significantly higher in larvae receiving Mp708 donor frass (p = 0.013), while OTU0008: *Kaistia* and OTU0009: *Rhodococcus* were elevated in those receiving Tx601 frass (p = 0.011 and p = 0.12, respectively).

### Influence of donor community on fall armyworm performance

**Figure 5:**
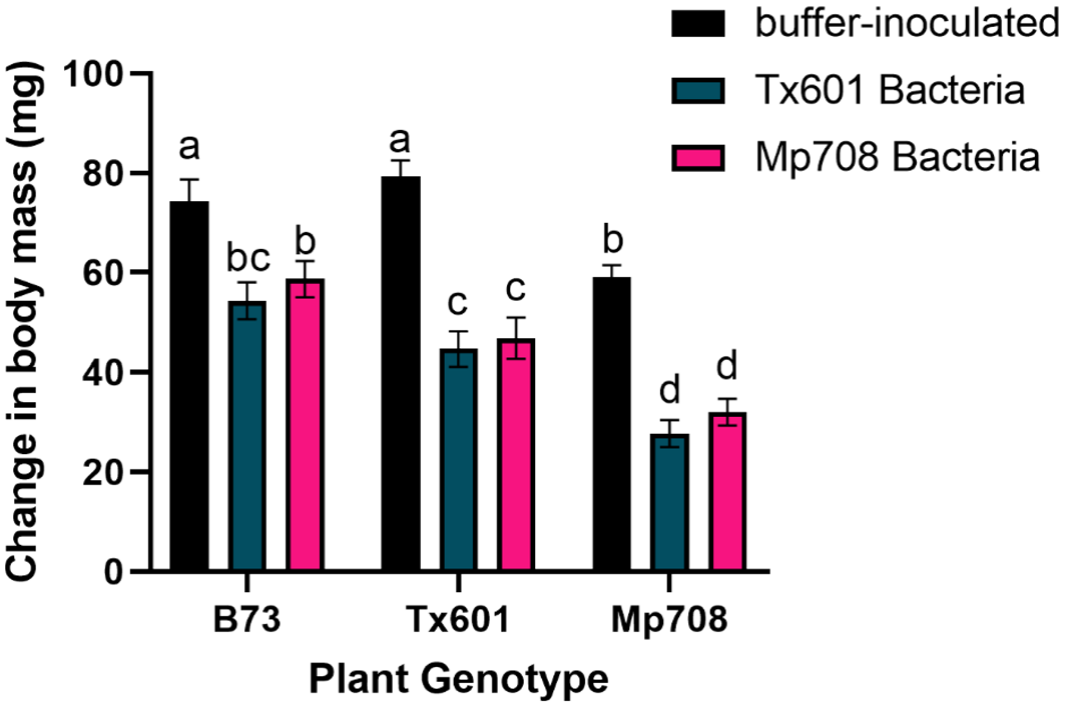
Effects of fecal transfer on recipient fall armyworm growth when fed on different maize genotypes. Different letters represent statistically significant differences (p < 0.05). Supplemental Figure 1: Non-metric multidimensional scaling of fall armyworm fed corn from the field using Bray–Curtis (A) and Jaccard (B) dissimilarities. Frass is included to compare with the guts of insects feeding on Tx601 and Mp708 foliage. Proportion of OTUs (C) and a heatmap (D) of OTUs illustrating the different assemblages produced by the frass compared to the gut tissues.

While we observed variation in the bacterial communities based on donor sources, they did not translate to differential effects on fall armyworm performance (Fig. 5). For the experiment, we observed a significant effect of the bacterial inoculation (F_1,142_ = 76.8; p < 0.001) and bioassay plant genotype (F_2,142_ = 19.8; p < 0.001) on larval performance, but only a marginally insignificant interaction between the two (F_2,142_ = 2.64; p = 0.075). For both inoculation treatments and across all bioassay plants, we observed reductions in growth of axenic larvae inoculated with fecal microbes compared to axenic larvae that were mock inoculated. Larvae inoculated with fecal microbes had growth reductions of 30%, 44%, and 55% compared to the axenic larvae fed on B73, Tx601, and Mp708 foliage, respectively.

## Discussion

Multiple factors influence gut microbial community assemblages of insect herbivores. Host immune recognition, immigration of new microbes and subsequent competition, as well as diet phytochemistry can alter microbial colonization and ultimate composition. Our results reveal the interconnectedness of the larval host environment, bacterial sources, and plant genotypes on ultimately shaping fall armyworm gut microbial communities. Three main findings arise from our data. First, these data support the notion that the insect gut environment can help shape microbial communities, and that colonization is not random. Second, our data show that different microbial mixtures, although initially very similar, can produce different assemblages in the herbivore gut. Finally, our results lend support to the fact that different plant genotypes can lead to distinct bacterial communities. These results also support prior experiments showing that gut bacteria can exert performance costs on the caterpillar, ultimately reducing growth^16^. Collectively, plants and their associated microbes can play instrumental roles in shaping lepidopteran communities and, in some instances, can elicit negative impacts on herbivore host growth.

Insects encounter a diversity of microbes in the environment, but only a subset can colonize and thrive in their gut, as reported here. The host appears to shape the community differently from the initial source of gut bacteria. Similar patterns have been observed for other insect species^21^. A subset of bacteria associated with fall armyworm frass are transient since some taxa (for ex. *Acinetobacter, Sphingobacterium, Flavobacterium*) were observed at moderate abundance in the frass (> 5%) but were at very low relative abundance in the recipients. In contrast, the most prevalent OTUs we detected here (i.e., *Enterococcus, Pseudomonas,* and Enterobacteriaceae) have been observed in other surveys in this system feeding on different plant substrates^7,16,32–34^. We also detected taxa that were uncommon or at low abundances in other studies of fall armyworm^7,32^, specifically *Paenibacillus* and *Kaistia*. While we are unclear about core functions of most gut bacteria in the fall armyworm system, it should be noted that *Paenibacillus* strains can be pathogens of arthropods^39^. Under controlled conditions where immigration of microbial populations is limited, some of the rarer taxa proliferated. We do not know the attributes that enable taxa to establish in lepidopteran gut systems. Some physiological components such as high pH tolerance and evasion of host immune responses are likely involved in mediating host-microbe interactions^40^. More studies are needed to fully parse determinants of colonization in these systems.

There is an increasing body of evidence that plants and their associated microbes can influence which taxa populate lepidopteran guts^4,41^. However, there are mixed reports regarding the effects of plant genotypes on gut bacterial communities. Some studies show that plant species and genotypes can structure microbial communities^15,42^, while others have documented minimal impacts^43^. We observed a differential impacts of plant genotype between our experiments with field-grown maize and laboratory conditions. When larvae consumed field-collected leaves, only a few dominant OTUs varied between larvae feeding on different maize genotypes and compositional differences were driven by less dominant taxa. Under controlled conditions, plant genotype emerged to influence community structure and membership. A likely explanation for this apparent contradiction is that fall armyworm feeding on field-grown maize experienced repeated introductions of bacterial taxa into their gut tissues, so the overall effects of plant genotype may have been dampened. Under controlled conditions, the patterns we observed were derived from a community reacting to the gut habitat and different microbial sources adapting to plant diets.

The maize genotypes used in our study possess different suites of plant defenses, but why the gut communities responded differently to these genotypes is unclear. These susceptible and resistant maize genotypes have been shown to elicit different immune responses in fall armyworm larvae^16,44^, but we only have a nascent understanding of the impact of fall armyworm immune responses on gut bacteria. We previously observed that fall armyworm bacteria can escape the gut and colonize the hemolymph, which ultimately reduces larval performance and increases mortality^16^. Our results support these prior findings, where gut bacterial communities significantly reduced larval growth on the different maize lines. Interestingly, the underlying changes in the bacterial communities did not substantially influence larval performance. It is possible that the transplants possess some core bacterial members have negative repercussions on the host insects when encountering these maize diets. Future work incorporating members isolated form this community or potentially more virulent pathogens within complex communities is needed to elucidate the mechanisms and functions of single bacterial isolates vs. multiple taxa in the community.

Animal feces can be a useful proxy for continuous, nondestructive assessment of gut microbes associated with hosts. Our results suggest that frass may not be a suitable proxy for reflecting the microbial communities present in the lepidopteran gut, and support other findings documenting a discordance between frass and caterpillar gut samples^43^. However, our findings are in contrast to other studies where caterpillar frass was a close representative of microbial taxa in the host gut^12,45^. This may be due to some caveats associated with the interpretation of our data. Since frass was pooled, stored at 4 ºC for five days until processed, and then suspended in buffer, this may introduce sampling and DNA extraction biases that could have contributed to our results. Ultimately, caution should be taken when using frass as a proxy for the gut microbial community in lepidopterans and, ideally, further validation of microbial compositions with paired gut and frass samples are needed.

One of the challenges of working with lepidopteran-microbial relationships reared in laboratory culture is that they seldom represent conspecifics collected in natural habitats^11,16^. Substantial changes in microbial composition often arise from using artificial diets, where gut microbial populations and communities of insects that feed on these diets can be skewed^38^. Connecting microbial interactions under controlled laboratory conditions to what occurs in natural populations can be inherently challenging. Frass homogenates may serve as an avenue for generating more natural assemblages of microbes, but this method contains potential drawbacks. For instance, there is a loss of experimental control using cultured populations or communities, however, using combinations of isolates with a full community transplant can mitigate this drawback. Additionally, there may be challenges in predicting how the community may react under the experimental conditions. Finally, we do not know how long and or how stable these communities may be under deep freeze, and how freeze–thaw cycles may impact cell viability. With these caveats in mind, frass homogenates should be explored as a future tool for introducing microbial complexity in the laboratory to evaluate multipartite interactions.

Interrelationships between insects, plants, and respective microbial partners can be incredibly complicated, especially as genotypic permutations are superimposed on studies. Here we showed how host plant genotype and inoculation sources can ultimately shape larval gut microbial communities and host phenotype. Currently, we do not know how fall armyworm mediates microbial population dynamics and community composition. Since these are not fully transient communities, it is likely that the gut environment plays an instrumental role in dictating whether specific community members can establish and proliferate. Future work is needed to understand microbial interactions for lepidopterans; incorporating the impacts of plants and variable diets in these equations is integral for understanding the physiological ecology of these systems.

## Methods

### Plant and insect sources

Fall armyworm eggs were obtained from Benzon Research (Carlisle, PA, USA). Immediately upon receipt, eggs were surface sterilized in 3% bleach for 5 min, rinsed twice in freshly autoclaved water, and dried in a laminar flow hood. Insects were then hatched *en masse* in a 250 mL sterile arena and maintained on sterilized wheat-germ based artificial diet at 25 °C until use in experiments. The autoclaved diet included all components of the previously described diet^46^, but lacked bactericidal antibiotics that may compromise establishment and/or responses of bacteria in subsequent experiments^38^.

We used three maize genotypes for our experiments: Mp708, which expresses a novel cysteine protease that degrades the fall armyworm’s peritrophic matrix, which is a barrier that protects the midgut epithelial cells from the food bolus^47,48^; Tx601, the fall armyworm susceptible parent of Mp708; and B73 which is a separate susceptible maize line. Seeds were planted in the field and in the greenhouse. Field-planted maize was grown at the Pennsylvania State University Russell E. Larson Agricultural Research Center at Rock Springs, PA until the V6-V8 stage. Field-grown maize were planted ~ 30 cm apart in adjacent rows in the same plot. Greenhouse plants were germinated in Promix-HP potting mix (Premier Tech Home and Garden, Ontario, Canada) under a 16:8 h L:D cycle at 27 °C. Plants were transplanted into a 3:1 ratio of field soil and potting mix until the V6 maize growing stage^49^.

### Experimental workflow and sample collection

Insect samples were collected at multiple intervals and included donor guts, frass from the donors, and the guts from the larval recipients at the end of the experiment. In order to initially test the effects of plant-associated microbes among different genotypes on herbivore gut microbes, we reared fall armyworm to fourth instar then fed them maize leaves grown in the field. Leaves were collected from the field and larvae were provided cut up leaves placed on 1% agar in 25 mL diet cups. Larvae were allowed to feed for 1 week, with frass being collected and pooled in a 50 mL conical tube held at 4 °C. After 1 week, larvae were anesthetized, surface sterilized in 70% ethanol, and their guts were dissected and immediately frozen in liquid nitrogen and stored at – 80 °C until DNA extraction.

We used frass pooled from insects feeding on different plant genotypes (donors) to provide to new, axenic recipients to test how different bacterial communities may populate and establish in controlled environments. We performed a washing procedure to reduce large frass particulates from the microbes present in the excrement using a series of centrifugation steps. To do this, 15 g of frass was suspended in 50 mL of ice-cold sterile phosphate buffered saline (pH 7.0; PBS) and centrifuged at 4 °C at 500×*g* for 30 min. Supernatant was removed and the resulting pellet was resuspended in 50 mL of sterile PBS and centrifuged with washes in between; this was repeated two additional times. After the three spins at 500×*g*, the pellet was resuspended and spun at 2500×*g* for 15 min. The pellet was resuspended in 5 mL of PBS, and 20% glycerol stocks were generated and stored at − 80 °C. Our preparations yielded bacterial densities of ~ 10^7^ colony forming units (CFUs) µL^−1^ after recovery from the glycerol stocks. These donor communities were constructed for insects consuming Tx601 and Mp708 and used to inoculate new insects (recipients). DNA was extracted from a portion of the mixed community used to inoculate the new larvae.

Recipient fall armyworm larvae were inoculated with frass bacterial communities following methods described previously^16,38^. Sterilized second instar fall armyworm were administered 10^7^ CFUs of the bacterial cocktail on artificial diet and allowed to feed for 24 h. After inoculation, larvae were weighed and provided gamma-irradiated leaves of greenhouse grown B73, Tx601, and Mp708 maize in sterile 22.5 mL containers. Plant leaves were cut into equal sized segments and sterilized with gamma irradiation in sealed plastic bags. Gamma rays were produced from radioactive Cobalt-60, which has two high energy gamma rays (1.17 MeV and 1.33 meV—average of 1.25 meV) and was performed at The Pennsylvania State University Radiation Science and Engineering Center Gamma Irradiation Facility. Sterilized leaves were stored at 4 °C and replaced every 1–2 days. Bioassays were conducted for 7 days, after which larvae were weighed and a randomized subset of larvae were selected to have their guts dissected to sequence bacterial communities. At the beginning and end of the experiment, a random portion of insects receiving a PBS-glycerol mock inoculation were assessed for their axenic status by plating onto nutrient-rich yeast tryptone medium. Additionally, a portion of the gamma-irradiated plant tissues were tested for bacteria by culturing on nutrient-rich media; we observed no detectable, viable microbes.

### Amplicon sequencing and analysis

DNA was extracted using the Quick-DNA Fecal/Soil Microbe Microprep Kit (Zymo Research, Irvine, CA, USA) according to the manufacturer’s instructions. 16S-rRNA V4 amplicons were generated using 515F and 806R primers^50,51^. PCRs were conducted using Platinum HotStart MasterMix (ThermoFisher Scientific, Waltham, MA, USA) with the following conditions: 94 °C for 3 min, 30 cycles of 94 °C for 45 s, 50 °C for 60 s, 72 °C for 90 s, and a final annealing hold of 72 °C for 10 min. Amplicon barcodes and Illumina-specific sequences were added with an additional round of PCR and sequenced with MiSeq V3 chemistry at the Pennsylvania State University Hershey Medical Campus Genomics Core Facility (Hershey, PA, USA).

Amplicon sequences were processed and binned into operational taxonomic units (OTUs) using mothur v.1.43.0^52^. Briefly, contigs were formed, trimmed, and aligned to the Silva SEED database (v132). Chimeric sequences were detected and removed from samples using VSEARCH as implemented in mothur. Sequences were classified using the RDP reference taxonomy training set (v16) and sequences that were classified as eukaryote, plastids, or “unknowns” were removed. We opted to use 97% similarity to bin OTUs. Samples were then randomly subset to 25,000 sequences per sample and the resulting OTU tables were used for the following downstream analyses.

### Statistical analyses

We performed a series of multi- and univariate analyses on the bacterial communities and individual OTUs. Visualization of the communities were conducted by non-metric multidimensional scaling (NMDS) using Bray–Curtis (relative abundance) and Jaccard (presence/absence) dissimilarities using PRIMER-E (v7). Permutation multivariate analysis of variance (PerMANOVA) was performed in PRIMER-E to test main and pairwise differences in treatments and groups using 999 model permutations. Alpha diversity metrics were computed in mothur and analyzed using an analysis of variance in R-studio v.1.2.5033 using the R v.3.6.3^53^. Visualization and clustering of individual OTUs were performed using log_2_ transformed values and Euclidean distances in the pheatmap package in R^54^. Differences in specific OTU relative abundances in the bioassay were performed using a nonparametric two-way aligned ranks transformation ANOVA using ARTools^55,56^. We performed post hoc contrasts to evaluate how plant genotypes affected bioassay microbial communities. Pairwise comparisons evaluating the influence of the bioassay plant on the microbial community were performed within each donor group using a Kruskal–Wallis test with a Holm correction. Larval growth bioassays were evaluated using an ANCOVA approach^57^, with bacterial inoculation and bioassay plants as fixed effects and initial mass as a covariate.

## Supplementary Information

Supplementary Information 1.

Supplementary Information 2.

Supplementary Information 3.

## Data Availability

Raw amplicon sequence data have been deposited in the NCBI Sequence Read Archive under accession number PRJNA658986.

## References

[1] Moran NA, Ochman H, Hammer TJ (2019). Evolutionary and ecological consequences of gut microbial communities. Annu. Rev. Ecol. Syst..

[2] Engel P, Moran NA (2013). The gut microbiota of insects—Diversity in structure and function. FEMS Microbiol. Rev..

[3] Douglas AE (2015). Multiorganismal insects: Diversity and function of resident microorganisms. Annu. Rev. Entomol..

[4] Paniagua Voirol LR, Frago E, Kaltenpoth M, Hilker M, Fatouros NE (2018). Bacterial symbionts in Lepidoptera: Their diversity, transmission, and impact on the host. Front. Microbiol..

[5] Mason CJ (2020). Complex relationships at the intersection of insect gut microbiomes and plant defenses. J. Chem. Ecol..

[6] Hammer TJ, Sanders JG, Fierer N (2019). Not all animals need a microbiome. FEMS Microbiol. Lett..

[7] Jones A, Mason C, Felton G, Hoover K (2019). Host plant and population source drive diversity of microbial gut communities in two polyphagous insects. Sci. Rep..

[8] Hammer TJ, Janzen DH, Hallwachs W, Jaffe SP, Fierer N (2017). Caterpillars lack a resident gut microbiome. Proc. Natl. Acad. Sci..

[9] Broderick NA, Raffa KF, Goodman RM, Handelsman J (2004). Census of the bacterial community of the gypsy moth larval midgut by using culturing and culture-independent methods. Appl. Environ. Microbiol..

[10] Shao Y, Arias-Cordero E, Guo H, Bartram S, Boland W (2014). In vivo Pyro-SIP assessing active gut microbiota of the cotton leafworm, *Spodoptera littoralis*. PLoS ONE.

[11] Priya NG, Ojha A, Kajla MK, Raj A, Rajagopal R (2012). Host plant induced variation in gut bacteria of *Helicoverpa armigera*. PLoS ONE.

[12] Mason CJ, Raffa KF (2014). Acquisition and structuring of midgut bacterial communities in gypsy moth (Lepidoptera: Erebidae) larvae. Environ. Entomol..

[13] Martemyanov VV (2016). Phenological asynchrony between host plant and gypsy moth reduces insect gut microbiota and susceptibility to *Bacillus thuringiensis*. Ecol. Evol..

[14] Chen B (2020). Gut microbiota metabolic potential correlates with body size between mulberry-feeding lepidopteran pest species. Pest Manag. Sci..

[15] Su’ad AY (2019). Host plant-dependent effects of microbes and phytochemistry on the insect immune response. Oecologia.

[16] Mason CJ (2019). Plant defenses interact with insect enteric bacteria by initiating a leaky gut syndrome. Proc. Natl. Acad. Sci..

[17] Staudacher H (2016). Variability of bacterial communities in the moth *Heliothis virescens* indicates transient association with the host. PLoS ONE.

[18] Ericsson AC, Personett AR, Turner G, Dorfmeyer RA, Franklin CL (2017). Variable colonization after reciprocal fecal microbiota transfer between mice with low and high richness microbiota. Front. Microbiol..

[19] Kreisinger J (2017). Temporal stability and the effect of transgenerational transfer on fecal microbiota structure in a long distance migratory bird. Front. Microbiol..

[20] Stappenbeck TS, Virgin HW (2016). Accounting for reciprocal host-microbiome interactions in experimental science. Nature.

[21] Mikaelyan A, Thompson CL, Hofer MJ, Brune A (2016). Deterministic assembly of complex bacterial communities in guts of germ-free cockroaches. Appl. Environ. Microbiol..

[22] Salem H, Florez L, Gerardo N, Kaltenpoth M (2015). An out-of-body experience: The extracellular dimension for the transmission of mutualistic bacteria in insects. Proc. R. Soc. Lond. B Biol. Sci..

[23] Powell JE, Martinson VG, Urban-Mead K, Moran NA (2014). Routes of acquisition of the gut microbiota of *Apis mellifera*. Appl. Environ. Microbiol..

[24] Brune A (2014). Symbiotic digestion of lignocellulose in termite guts. Nat. Rev. Microbiol..

[25] Chen B (2018). Gut bacterial and fungal communities of the domesticated silkworm (*Bombyx mori*) and wild mulberry-feeding relatives. ISME J..

[26] Hannula S, Zhu F, Heinen R, Bezemer T (2019). Foliar-feeding insects acquire microbiomes from the soil rather than the host plant. Nat. Commun..

[27] Montezano DG (2018). Host plants of *Spodoptera frugiperda* (Lepidoptera: Noctuidae) in the Americas. Afr. Entomol..

[28] Day R (2017). Fall armyworm: Impacts and implications for Africa. Outlooks Pest Manag..

[29] Visôtto LE, Oliveira MGA, Guedes RNC, Ribon AOB, Good-God PIV (2009). Contribution of gut bacteria to digestion and development of the velvetbean caterpillar, *Anticarsia gemmatalis*. J. Insect Physiol..

[30] Xiang H (2006). Microbial communities in the larval midgut of laboratory and field populations of cotton bollworm (*Helicoverpa armigera*). Can. J. Microbiol..

[31] Tang X (2012). Complexity and variability of gut commensal microbiota in polyphagous lepidopteran larvae. PLoS ONE.

[32] Gomes AFF, Omoto C, Cônsoli FL (2020). Gut bacteria of field-collected larvae of *Spodoptera frugiperda* undergo selection and are more diverse and active in metabolizing multiple insecticides than laboratory-selected resistant strains. J. Pest Sci..

[33] Acevedo FE (2017). Fall armyworm-associated gut bacteria modulate plant defense responses. Mol. Plant-Microbe Interact..

[34] Gichuhi J (2020). Diversity of fall armyworm, *Spodoptera fugiperda* and their bacterial community in Kenya. PeerJ.

[35] Wagner MR, Busby PE, Balint-Kurti P (2020). Analysis of leaf microbiome composition of near-isogenic maize lines differing in broad-spectrum disease resistance. New Phytol..

[36] Naveed M, Mitter B, Reichenauer TG, Wieczorek K, Sessitsch A (2014). Increased drought stress resilience of maize through endophytic colonization by *Burkholderia phytofirmans* PsJN and *Enterobacter* sp. FD17. Environ. Exp. Bot..

[37] Keshri J (2018). Microbiome dynamics during ensiling of corn with and without *Lactobacillus plantarum* inoculant. Appl. Microbiol. Biotechnol..

[38] Mason CJ (2020). Diet influences proliferation and stability of gut bacterial populations in herbivorous lepidopteran larvae. PLoS ONE.

[39] Chan QWT, Melathopoulos AP, Pernal SF, Foster LJ (2009). The innate immune and systemic response in honey bees to a bacterial pathogen, *Paenibacillus larvae*. BMC Genomics.

[40] Mazumdar, T. *et al.* Survival strategies of *Enterococcus mundtii* in the gut of *Spodoptera littoralis*: A live report. *bioRxiv*. 10.1101/2020.02.03.932053 (2020).

[41] Mason CJ, Jones AG, Felton GW (2018). Co-option of microbial associates by insects and their impact on plant–folivore interactions. Plant Cell Environ..

[42] Mason CJ, Rubert-Nason KF, Lindroth RL, Raffa KF (2014). Aspen defense chemicals influence midgut bacterial community composition of gypsy moth. J. Chem. Ecol..

[43] Chaturvedi S, Rego A, Lucas LK, Gompert Z (2017). Sources of variation in the gut microbial community of *Lycaeides melissa* caterpillars. Sci. Rep..

[44] Fescemyer HW (2013). Maize toxin degrades peritrophic matrix proteins and stimulates compensatory transcriptome responses in fall armyworm midgut. Insect Biochem. Mol. Biol..

[45] Hammer TJ, McMillan WO, Fierer N (2014). Metamorphosis of a butterfly-associated bacterial community. PLoS ONE.

[46] Chippendale GM (1970). Metamorphic changes in haemolymph and midgut proteins of the southwestern corn borer, *Diatraea grandiosella*. J. Insect Physiol..

[47] Pechan T, Cohen A, Williams WP, Luthe DS (2002). Insect feeding mobilizes a unique plant defense protease that disrupts the peritrophic matrix of caterpillars. Proc. Natl. Acad. Sci..

[48] Mohan S (2006). Degradation of the *S. frugiperda* peritrophic matrix by an inducible maize cysteine protease. J. Insect Physiol..

[49] Tsuji GY, Hoogenboom G, Thornton PK (2013). Understanding Options for Agricultural Production.

[50] Apprill A, McNally S, Parsons R, Weber L (2015). Minor revision to V4 region SSU rRNA 806R gene primer greatly increases detection of SAR11 bacterioplankton. Aquat. Microb. Ecol..

[51] Parada AE, Needham DM, Fuhrman JA (2016). Every base matters: Assessing small subunit rRNA primers for marine microbiomes with mock communities, time series and global field samples. Environ. Microbiol..

[52] Schloss PD (2009). Introducing mothur: Open-source, platform-independent, community-supported software for describing and comparing microbial communities. Appl. Environ. Microbiol..

[53] R Core Team. R: A Language and Environment for Statistical Computing. (2020).

[54] Kolde, R. pheatmap: Pretty Heatmaps. R package version 1.0.12. https://CRAN.R-project.org/package=pheatmap (2018).

[55] Kay, M. & Wobbrock, J. ARTool: Aligned Rank Transform for Nonparametric Factorial ANOVAs. 10.5281/zenodo.594511, R package version 0.10.7, https://github.com/mjskay/ARTool (2020).

[56] Wobbrock, J., Findlater, L., Gergle, D., & Higgins, J.. The Aligned Rank Transform for Nonparametric Factorial Analyses Using Only ANOVA Procedures. In *Proceedings of the ACM Conference on Human Factors in Computing Systems* (CHI '11), 143–146.(2011).

[57] Raubenheimer D, Simpson SL (1992). Analysis of covariance: An alternative to nutritional indices. Entomol. Exp. Appl..

