# Supplementary material for: Effects of maize (Zea mays) genotypes and microbial sources in shaping fall armyworm (Spodoptera frugiperda) gut bacterial communities
Source: Sci Rep. 2021 Feb 24;11:4429. doi: 10.1038/s41598-021-83497-2 (PMC7904771; doi:10.1038/s41598-021-83497-2)
Supplement: Supplementary file 1 — Supplementary Information 1. [file 41598_2021_83497_MOESM1_ESM.docx]

**Supplemental Figure 1:** Non-metric multidimensional scaling of fall armyworm fed corn from the field using Bray-Curtis (A) and Jaccard (B) dissimilarities. Frass is included to compare with the guts of insects feeding on Tx601 and Mp708 foliage. Proportion of OTUs (C) and a heatmap (D) of OTUs illustrating the different assemblages produced by the frass compared to the gut tissues.
