# Supplementary figures and images for: Effects of maize (Zea mays) genotypes and microbial sources in shaping fall armyworm (Spodoptera frugiperda) gut bacterial communities
Source: Sci Rep. 2021 Feb 24;11:4429. doi: 10.1038/s41598-021-83497-2 (PMC7904771; doi:10.1038/s41598-021-83497-2)

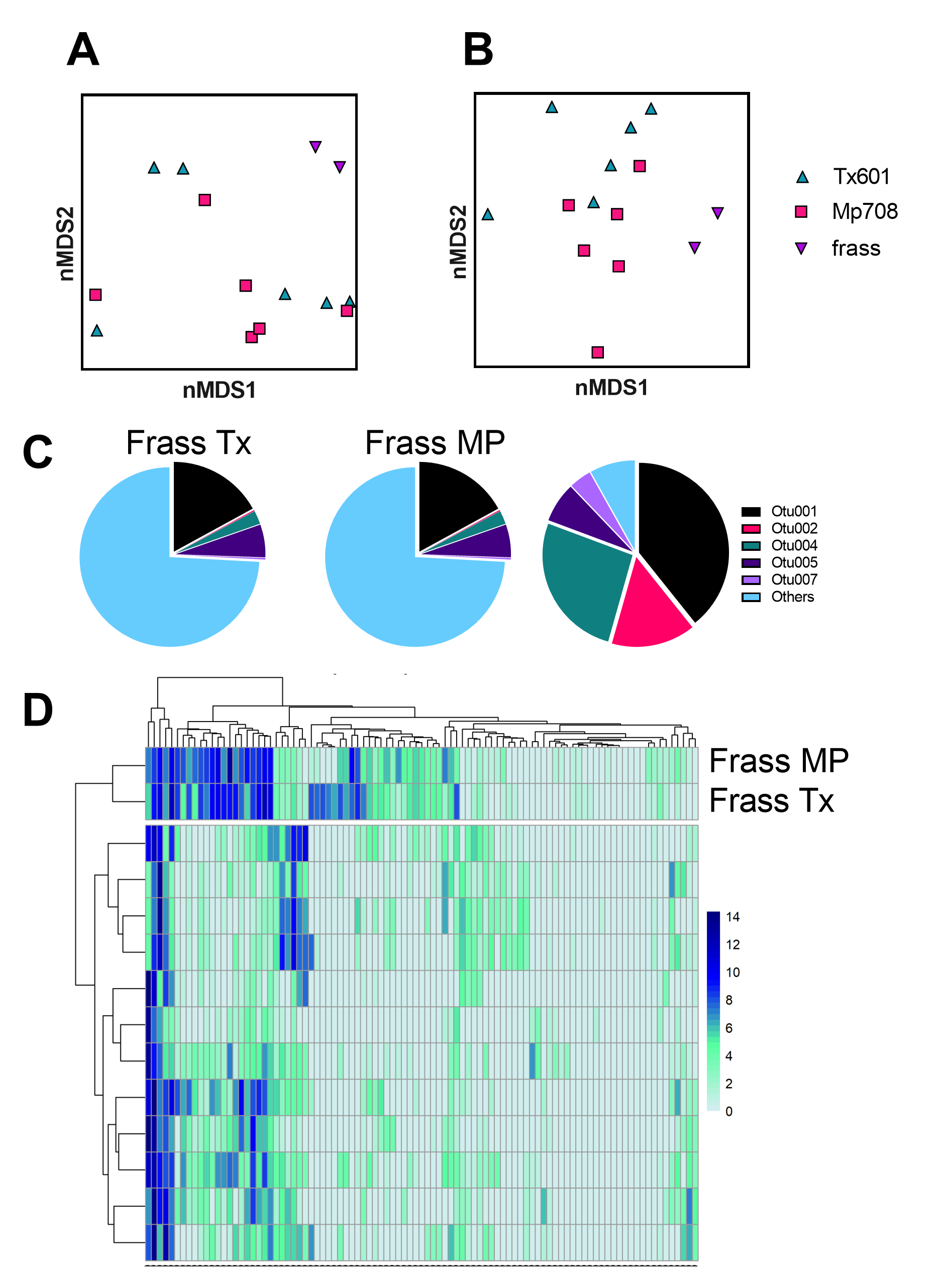

Supplement: Supplementary file 2 — Supplementary Information 2. [file 41598_2021_83497_MOESM2_ESM.tif]
