# Supplementary material for: Effects of maize (Zea mays) genotypes and microbial sources in shaping fall armyworm (Spodoptera frugiperda) gut bacterial communities
Source: Sci Rep. 2021 Feb 24;11:4429. doi: 10.1038/s41598-021-83497-2 (PMC7904771; doi:10.1038/s41598-021-83497-2)
Supplement: Supplementary file 3 — Supplementary Information 3. [file 41598_2021_83497_MOESM3_ESM.docx]

**Title**: Effects of maize (*Zea mays*) genotypes and microbial sources in shaping fall armyworm (*Spodoptera frugiperda*) gut bacterial communities

**Authors**: Charles J. Mason, Kelli Hoover, Gary W. Felton

Department of Entomology, The Pennsylvania State University, University Park, PA, 16802

**Address correspondence**: Charles Mason – cjm360@psu.edu

| Supplemental Table 1: Alpha diversity metrics of OTUs fall armyworm midguts that fed on field-grown maize (mean±s.e). No significant differences were observed between proportions using an ANOVA | | | | | |
| --- | --- | --- | --- | --- | --- |
|  | Plant Genotype | | | ANOVA | |
| Metric: | B73 | Tx601 | Mp708 | F-value | P-value |
| OTU Richness | 105±10.7 | 104.5±10.8 | 92.5±8.2 | 0.501 | 0.616 |
| Chao1 | 156.9±23.4 | 170.2±18.7 | 152.9±21.5 | 0.208 | 0.814 |
| Shannon | 1.19±0.23 | 1.24±0.11 | 1.15±0.11 | 0.75 | 0.928 |
| 1/Simpson | 2.14±0.37 | 2.17±0.19 | 2.36±0.17 | 0.182 | 0.835 |

| Supplemental Table 2: Most abundant OTUs (proportion) associated with fall armyworm fed on field-grown maize (mean±s.e). No significant differences were observed between proportions using an align rank transformed ANOVA. | | | | | |
| --- | --- | --- | --- | --- | --- |
|  | Plant genotype: | | | Aligned Rank Transformed ANOVA | |
| Operational Taxonomic Unit: | B73 | Tx601 | Mp708 | f-value | p-value |
| OTU001 Unclassified Enterobacteriaceae | 0.42±0.09 | 0.35±0.12 | 0.40±0.14 | 0.083 | 0.920 |
| OTU002: Enterococcus | 0.02±0.11 | 0.16±0.13 | 0.27±0.10 | 0.700 | 0.512 |
| OTU004: Pseudomonas | 0.37±0.09 | 0.24±0.11 | 0.19±0.11 | 0.730 | 0.498 |
| OTU005: Neorhizobium | 0.04±0.07 | 0.10±0.06 | 0.08±0.02 | 0.021 | 0.980 |
| OTU007: Unclassified Enterobacteriaceae | 0.06±0.01 | 0.05±0.04 | 0.01±0.02 | 2.59 | 0.108 |

| Supplemental Table 3: OTU alpha diversity metrics associated with fall armyworm frass concentrated and donated to axenic recipient larvae | | |
| --- | --- | --- |
|  | Donor Genotype | |
| Metric: | Tx601 | Mp708 |
| OTU Richness | 197 | 178 |
| Chao1 | 317.1 | 302.2 |
| Shannon | 2.98 | 2.59 |
| 1/Simpson | 10.9 | 6.06 |

| Supplemental Table 4: Predominant OTUs associated with fall armyworm concentrated frass. Raw reads were out of 25000, and proportion is relative abundance | | | | |
| --- | --- | --- | --- | --- |
|  | Raw Read | | Proportion | |
| OTU with RDP classification (confidence level) | Tx601 | Mp708 | Tx601 | Mp708 |
| OTU0001:Enterobacteriaceae_unclassified(100) | 4256 | 4662 | 0.17024 | 0.18648 |
| OTU0002:Enterococcus(99) | 45 | 133 | 0.0018 | 0.00532 |
| OTU0003:Paenibacillus(99) | 0 | 4 | 0 | 0.00016 |
| OTU0004:Pseudomonas(100) | 608 | 385 | 0.02432 | 0.0154 |
| OTU0005:Neorhizobium(95) | 1474 | 871 | 0.05896 | 0.03484 |
| OTU0007:Enterobacteriaceae_unclassified(95) | 77 | 154 | 0.00308 | 0.00616 |
| OTU0008:Kaistia(100) | 52 | 46 | 0.00208 | 0.00184 |
| OTU0009:Rhodococcus(100) | 232 | 46 | 0.00928 | 0.00184 |
| OTU0010:Leuconostoc(100) | 0 | 2 | 0 | 0.00008 |
| OTU0011:Stenotrophomonas(100) | 778 | 476 | 0.03112 | 0.01904 |
| OTU0012:Ochrobactrum(99) | 37 | 62 | 0.00148 | 0.00248 |
| OTU0014:Paenibacillus(100) | 2 | 109 | 0.00008 | 0.00436 |
| OTU0015:Flavobacterium(100) | 531 | 8596 | 0.02124 | 0.34384 |
| OTU0016:Aureimonas(97) | 33 | 9 | 0.00132 | 0.00036 |
| OTU0017:Pseudomonas(100) | 5 | 24 | 0.0002 | 0.00096 |
| OTU0018:Acinetobacter(100) | 3743 | 949 | 0.14972 | 0.03796 |
| OTU0019:Rhizobium(100) | 230 | 2 | 0.0092 | 0.00008 |
| OTU0020:Sphingobacterium(100) | 4079 | 1018 | 0.16316 | 0.04072 |
| OTU0021:Pseudomonas(99) | 1303 | 818 | 0.05212 | 0.03272 |

| \| Supplemental Table 5: Pairwise perMANOVA *P*-values of fall armyworm gut communities receiving donor frass and fed different bioassay plants. Pairwise comparisons were conducted within a donor group. \| \| \| \| \| \| \| \| --- \| --- \| --- \| --- \| --- \| --- \| --- \| \| Bray-Curtis: \| Donor Tx601 \| \| \| Donor Mp708 \| \| \| \| B73 \| Tx601 \| Mp708 \| B73 \| Tx601 \| Mp708 \| \| B73 \| x \|  \|  \| x \|  \|  \| \| Tx601 \| 0.165 \| x \|  \| 0.16 \| x \|  \| \| Mp708 \| **0.012** \| **0.001** \| x \| 0.579 \| 0.061 \| x \| \| Jaccard: \| Donor Tx601 \| \| \| Donor Mp708 \| \| \| \| B73 \| Tx601 \| Mp708 \| B73 \| Tx601 \| Mp708 \| \| B73 \| x \|  \|  \|  \|  \|  \| \| Tx601 \| **0.01** \| **x** \|  \| 0.121 \|  \|  \| \| Mp708 \| **0.002** \| **0.001** \| x \| **0.023** \| **0.016** \|  \|   Supplemental Table 6: Influence of donor source (frass from Tx601 or Mp708-fed larvae) and bioassay plant genotype on alpha diversity metrics of OTUs in guts of fall armyworm. Significant P-values are bolded. | | | | | | | | | |
| --- | --- | --- | --- | --- | --- | --- | --- | --- | --- | --- | --- | --- | --- | --- | --- | --- | --- | --- | --- | --- | --- | --- | --- | --- | --- | --- | --- | --- | --- | --- | --- | --- | --- | --- | --- | --- | --- | --- | --- | --- | --- | --- | --- | --- | --- | --- | --- | --- | --- | --- | --- | --- | --- | --- | --- | --- | --- | --- | --- | --- | --- | --- | --- | --- | --- | --- | --- | --- | --- | --- | --- | --- | --- | --- | --- | --- | --- | --- | --- | --- | --- | --- | --- | --- |
|  | Tx601 - donor | | | Mp708 - donor | | | 2-way ANOVA effects  (p-values) | | |
| Metric: | B73 | Tx601 | Mp708 | B73 | Tx601 | Mp708 | Source | Bioassay | Interaction |
| OTU Richness | 61.6±10.7 | 40.2±2.8 | 56±3.2 | 45.5±3.8 | 73.3±8.5 | 50.33±4.3 | **0.01** | 0.967 | **0.02** |
| Chao1 | 139.7±19.8 | 119.4±17.8 | 140.1±14.7 | 91.0±11.4 | 176.2±33.5 | 112.63±7.8 | 0.08 | 0.288 | **0.05** |
| Shannon | 1.07±0.11 | 0.63±0.1 | 1.36±0.11 | 0.88±0.10 | 1.38±0.17 | 1.06±0.15 | **0.049** | 0.099 | **<0.001** |
| 1/Simpson | 2.41±0.27 | 1.53±0.14 | 2.99±0.38 | 1.89±0.16 | 3.3±0.48 | 2.46±0.44 | **0.04** | 0.207 | **0.005** |

| Supplemental Table 7: Aligned rank transformed ANOVA table. Microbe source indicates the donor frass the recipient larvae received, and bioassay plant is the experimental plant genotype. | | | |
| --- | --- | --- | --- |
| OTU with RDP classification (confidence level) | Microbe Source | Bioassay Plant | Interaction |
| OTU0001:Enterobacteriaceae_unclassified(100) | **F=21.4; p<0.001** | F=0.92; p=0.406 | **F=6.33; p=0.004** |
| OTU0002:Enterococcus(99) | **F=15.1; p<0.001** | F=1.48; p=0.237 | F=1.55; p=0.222 |
| OTU0003:Paenibacillus(99) | **F=45.1; p<0.001** | **F=5.60; p=0.006** | **F=5.44; p=0.007** |
| OTU0004:Pseudomonas(100) | F=1.39; p=0.244 | F=1.84; p=0.170 | F=0.07; p=0.931 |
| OTU0005:Neorhizobium(95) | F=0.01; p=0.932 | F=0.44; p=0.643 | F=2.81; p=0.071 |
| OTU0007:Enterobacteriaceae_unclassified(95) | **F=14.7; p<0.001** | F=0.45; p=0.640 | **F=4.05; p=0.024** |
| OTU0008:Kaistia(100) | **F=11.3; p=0.002** | **F=12.0; p<0.001** | **F=10.7; p<0.001** |
| OTU0009:Rhodococcus(100) | **F=19.9; p<0.001** | **F=8.30; p<0.001** | **F=20.5; p<0.001** |
| OTU0010:Leuconostoc(100) | **F=20.7; p<0.001** | **F=4.45; p=0.017** | **F=4.68; p=0.014** |
| OTU0011:Stenotrophomonas(100) | **F=15.2; p<0.001** | F=1.42; p=0.251 | **F=3.79; p=0.030** |
| OTU0012:Ochrobactrum(99) | **F=4.72; p=0.035** | **F=7.80; p=0.001** | F=1.14; p=0.328 |
| OTU0014:Paenibacillus(100) | **F=37.4; p<0.001** | **F=9.30; p<0.001** | **F=10.6; p<0.001** |
| OTU0015:Flavobacterium(100) | not tested < 1 read per sample | | |
| OTU0016:Aureimonas(97) | **F=32.0; p<0.001** | **F=4.50; p=0.17** | **F=11.8; p<0.001** |
| OTU0017:Pseudomonas(100) | not tested < 4 reads per sample | | |
| OTU0018:Acinetobacter(100) | **F=10.0; p=0.003** | **F=4.23; p=0.020** | **F=11.5; p<0.001** |
| OTU0019:Rhizobium(100) | **F=24.8; p<0.001** | F=3.1; p=0.054 | F=2.72; p=0. 076 |
| OTU0020:Sphingobacterium(100) | not tested < 1 read per sample | | |
| OTU0021:Pseudomonas(99) | F=0.27; p=0.605 | F=1.10; p=0.342 | F=0.80; p=0.456 |

| Supplemental Table 8: Relative abundances (mean ± se) of OTUs populating fall armyworm guts inoculated with Tx601 or Mp708 frass and fed gamma-irradiated B73, Tx601, or Mp708 maize. Contrasts were conducted using Wilcoxon tests with an FDR correction within a donor group, and letters represent significant differences within that group. “nd” indicates not detected in any samples. Accompanying overall p-values are provided in Supplemental Table 7. | | | | | | |
| --- | --- | --- | --- | --- | --- | --- |
|  | Tx601 Donor | | | Mp708 Donor | | |
| OTU with RDP classification (confidence level) | B73 | Tx601 | Mp708 | B73 | Tx601 | Mp708 |
| OTU0001:Enterobacteriaceae_unclassified(100) | 0.67±0.06^ab^ | 0.81±0.04^a^ | 0.49±0.08^b^ | 0.30±0.09 | 0.27±0.08 | 0.48±0.09 |
| OTU0002:Enterococcus(99) | 0.16±0.05 | 0.11±0.04 | 0.19±0.06 | 0.40±0.07 | 0.29±0.04 | 0.29±0.1 |
| OTU0003:Paenibacillus(99) | 0.01±0.01 | 0.01±0.01 | 0.01±0.01 | 0.11±0.05^AB^ | 0.29±0.08^A^ | 0.09±0.04^B^ |
| OTU0004:Pseudomonas(100) | 0.0061±0.0018 | 0.0052±0.0015 | 0.0124±0.0028 | 0.0145±0.0066 | 0.0096±0.0027 | 0.0178±0.0067 |
| OTU0005:Neorhizobium(95) | 0.006±0.005 | 0.014±0.0121 | 0.0001±0.0001 | 0.0003±0.0003 | nd | 0.0196±0.0183 |
| OTU0007:Enterobacteriaceae_unclassified(95) | 0.001±0.0004 | 0.0024±0.001 | 0.0009±0.0002 | 0.0636±0.0634 | 0.0028±0.0023 | 0.0022±0.0012 |
| OTU0008:Kaistia(100) | 0.0101±0.0065^b^ | 0.0161±0.0138^b^ | 0.0868±0.0159^a^ | 0.0268±0.0147^A^ | nd^B^ | 0.0235±0.0106^A^ |
| OTU0009:Rhodococcus(100) | 0.0126±0.0055^b^ | 0.0095±0.0045^b^ | 0.1371±0.0373^a^ | Nd^B^ | 0.0206±0.0128^A^ | 0.0091±0.0058^A^ |
| OTU0010:Leuconostoc(100) | 0.0004±0.0003^a^ | Nd^b^ | Nd^b^ | 0.0615±0.0398 | 0.0154±0.0063 | nd |
| OTU0011:Stenotrophomonas(100) | 0.0811±0.0593 | 0.002±0.0012 | 0.0025±0.0012 | 0.0043±0.0024 | 0.0001±0 | 0.0005±0.0002 |
| OTU0012:Ochrobactrum(99) | 0.0015±0.001^b^ | 0.0116±0.0115^b^ | 0.0373±0.0206^a^ | 0.0004±0.0004^B^ | 0.0169±0.0102^AB^ | 0.0619±0.0284^A^ |
| OTU0014:Paenibacillus(100) | nd | nd | nd | 0.0099±0.0083 | 0.0215±0.0078 | 0.0011±0.0005 |
| OTU0016:Aureimonas(97) | 0.0001±0.0001^b^ | 0.0004±0.0003^ab^ | 0.0187±0.0113^a^ | nd | nd | 0.0001±0.0001 |
| OTU0018:Acinetobacter(100) | Nd^b^ | Nd^b^ | 0.0006±0.0003^a^ | 0.0002±0.0001 | nd | nd |
| OTU0019:Rhizobium(100) | 0.0184±0.0093 | 0.0066±0.0056 | 0.0056±0.0041 | nd | 0.0001±0.0001 | nd |
| OTU0021:Pseudomonas(99) | 0.0022±0.0014 | 0.0006±0.0003 | 0.0005±0.0003 | 0.0021±0.0021 | 0.0001±0 | 0.0001±0 |

| Supplemental Table 9: Relative abundances (mean ± se) of OTUs populating fall armyworm guts consuming Mp708. Comparisons were conducted using Wilcoxon between donor group and significant differences are bolded. Accompanying overall p-values are provided in Supplemental Table 7. | | | |
| --- | --- | --- | --- |
| OTU with RDP classification (confidence level) | Tx601 donor | Mp708 donor | p-value |
| OTU0001:Enterobacteriaceae_unclassified(100) | 0.49±0.08 | 0.48±0.09 | 0.97 |
| OTU0002:Enterococcus(99) | 0.19±0.06 | 0.29±0.1 | 0.54 |
| OTU0003:Paenibacillus(99) | 0.01±0.01 | 0.09±0.04 | **0.013** |
| OTU0004:Pseudomonas(100) | 0.0124±0.0028 | 0.0178±0.0067 | 0.92 |
| OTU0005:Neorhizobium(95) | 0.0001±0.0001 | 0.0196±0.0183 | 0.79 |
| OTU0007:Enterobacteriaceae_unclassified(95) | 0.0009±0.0002 | 0.0022±0.0012 | 0.77 |
| OTU0008:Kaistia(100) | 0.0868±0.0159 | 0.0235±0.0106 | **0.011** |
| OTU0009:Rhodococcus(100) | 0.1371±0.0373 | 0.0091±0.0058 | **0.012** |
| OTU0010:Leuconostoc(100) | nd | nd | not tested |
| OTU0011:Stenotrophomonas(100) | 0.0025±0.0012 | 0.0005±0.0002 | 0.244 |
| OTU0012:Ochrobactrum(99) | 0.0373±0.0206 | 0.0619±0.0284 | 0.814 |
| OTU0014:Paenibacillus(100) | nd | 0.0011±0.0005 | **0.022** |
| OTU0016:Aureimonas(97) | 0.0187±0.0113 | 0.0001±0.0001 | **0.025** |
| OTU0018:Acinetobacter(100) | 0.0006±0.0003 | nd | **0.037** |
| OTU0019:Rhizobium(100) | 0.0056±0.0041 | nd | **0.003** |
| OTU0021:Pseudomonas(99) | 0.0005±0.0003 | 0.0001±0 | 0.999 |
